# miR-373-3p Regulates the Proliferative and Migratory Properties of Human HTR8 Cells via SLC38A1 Modulation

**DOI:** 10.1155/2022/6582357

**Published:** 2022-06-28

**Authors:** Lu Chen, Hong Wen, Yuhang Zhu, Fangfang Wang, Li Zhao, Qiongxin Liang, Fan Qu

**Affiliations:** Department of Obstetrics, Women's Hospital, Zhejiang University School of Medicine, Hangzhou, China

## Abstract

The genetic pathogenesis of selective intrauterine growth restriction (sIUGR) remains elusive, with evidence suggesting an important role of epigenetic factors such as microRNAs. In this study, we explored the relevance of miR-373-3p to the occurrence of sIUGR. Hypoxia enhanced the levels of miR-373-3p and hypoxia-inducible factor (HIF)-1*α*, while *HIF-1α* knockdown not only boosted the migration and proliferation of HTR8 cells but also suppressed the hypoxia-induced upregulation of miR-373-3p and SLC38A1. By contrast, HIF-1*α* overexpression induced miR-373-3p downregulation and SLC38A1 upregulation, reducing cell growth and migration, which could be reversed by a miR-373-3p inhibitor. Importantly, the miR-373-3p inhibitor and mimic reproduced phenomena similar to those induced by HIF-1*α* downregulation and overexpression, respectively (including altered SLC38A1 expression, mTOR activation, cell growth, and migration). Mechanistically, the miRNA regulated cell behaviors and related mTOR signaling by targeting SLC38A1 expression through an interaction with the 3′-untranslated region of SLC38A1. The placental tissues of smaller sIUGR fetuses exhibited miR-373-3p and HIF-1*α* upregulation, SLC38A1 downregulation, and activated mTOR. Overall, miR-373-3p appears to restrict the growth and migration of HTR8 trophoblast cells by targeting SLC38A1, as observed in the placental tissues associated with smaller sIUGR fetuses, and it could have utility in the diagnosis and treatment of this disorder.

## 1. Introduction

Selective intrauterine growth restriction (sIUGR) occurs in approximately 10%–15% of monochorionic twins and frequently causes perinatal morbidity and mortality [1, 2]. The mechanisms underlying this disorder remain unclear because monochorionic twins originate from the same zygote and have identical genomes [3, 4]. Considering the contribution of unequal placental sharing and vascular anastomoses to inconsistent fetal development [4–6], it is reasonable to speculate that the pathogenesis may be associated with the transportation of nutrients like amino acids. The sodium-coupled neutral amino acid transporter 1 (SLC38A1), an important amino acid transporter for energy metabolism and cell physiology, has key roles in neurotransmission, embryonic development, and cancer [7–10]. Indeed, SLC38A1 has confirmed roles in mouse placental development [7–10] and cancer cell growth and migration [7–10]. However, no research has examined its relevance to the pathogenesis of sIUGR.

The identical genomes of monochorionic twins have tended to preclude a major influence of genetic information on sIUGR progression, although this does hint at the potential significant roles of epigenetic factors. MicroRNAs (miRNAs) are short 21–25 nucleotide noncoding RNA molecules that regulate many cellular processes by causing gene-level alterations on binding to the 3′-untranslated region (3′-UTR) of a target gene [3, 11]. A growing body of evidence supports the use of miRNAs as potential biomarkers for pregnancy-associated disorders [12–16], with different or abnormal miR-210-3p and miR-338-5p expressions already linked to placental dysfunction in sIUGR [3, 17–19]. Whether other miRNAs have cognate roles in the pathogenesis of sIUGR requires further exploration.

Research indicates that miRNAs play key roles in physiological and pathological embryo implantation and development, and as such could represent diagnostic and therapeutic targets in pregnancy-related disorders [20, 21]. The miRNAs secreted by human embryos could serve as biomarkers in assisted reproductive technology [22], and evidence suggests that the expression profiles of miRNA in blastocoel fluid affect blastocyst quality [23]. By regulating target genes, miR-373-3p of the miRNA-371-372-373 family has been implicated in the development of multiple cancers [24–28]. Notably, a recent study also described the involvement of miR-373-3p in a pregnancy disorder, showing that it significantly impaired the migratory and invading capability of placental trophoblast cells by targeting CD44 and radixin [13]. The role of miR-373-3p and of its potential target SLC38A1 in the pathogenesis of sIUGR has not yet been explored by bioinformatic analysis.

In this study, we examine the effects of miR-373-3p and SLC38A1 on the phenotypes of a trophoblastic cell line exposed to hypoxia, mimicking the in vivo environment of sIUGR [29–32]. Our findings confirm the link between those molecules and show that miR-373-3p restricts the growth and migration of human trophoblastic cells by modulating SLC38A1.

## 2. Materials and Methods

### 2.1. Tissue Samples

sIUGR was defined by the International Society of Ultrasound in Obstetrics and Gynaecology (ISUOG) clinical guideline as the estimated fetal body weight (EFBW) below the 10th percentile in the smaller twin and the inter-twin EFBW discordance greater than 25%, in the absence of twin-to-twin transfusion syndrome or twin-anemia polycythemia sequence [33]. The inter-twin EFBW discordance was calculated using the formula (*A* − *B*) × 100/*A*, where *A* is the EFBW of the larger fetus and *B* is the EFBW of the sIUGR fetus. After delivery, sIUGR was further confirmed when birthweight of one twin was < 10th percentile. The definitions of control group were (1) monochorionic twin pregnancies and (2) both fetuses having normal estimated fetal weight and normal birthweight after delivery.

The territories of smaller twin and larger twin were recognized by their amniotic membranous septum and vascular distribution. The placenta tissues around the individual insertion region for each umbilical cord were collected within 30 min after delivery for each recruited case. The tissue was excised from inside the placental lobules, avoiding both the maternal surface and the amniotic membrane. At least 3 pieces of placentae were collected within it is territory for both twins. After that, chorions were divided from the decidua and amnion of placentae. Only chorions were used for the experiments.

Ten placental tissues from normal and sIUGR twins with high or low weight (*n* =5 for each group) were exploited to assess the mRNA and protein content of miR-373-3p and SLC38A1 by quantitative real-time PCR (qRT-PCR) and western blot. All participants signed informed consent forms. The independent ethics committee of Zhejiang University School of Medicine approved the current research, which complied with the Declaration of Helsinki.

### 2.2. Cell Culture

Placenta-derived human trophoblastic HTR8 cells were bought from the cell bank of Shanghai Biology Institute (Shanghai, P.R. China). These were maintained in Dulbecco's modified Eagle medium (Trueline, Kaukauna, WI, USA) with 10% fetal bovine serum (Thermo Fisher Scientific), 2 mM l-glutamine, and 1% penicillin/streptomycin (Solarbio, Beijing, P.R. China) in an incubator with a 5% CO_2_ atmosphere and a temperature of 37°C.

### 2.3. Quantitative Real-Time PCR

Total RNAs extracted from tissues or cells with TRIzol Reagent (1596-026, Invitrogen, Waltham, MA, USA) were subjected to reverse-transcription into cDNA using a commercial kit (#K1622, Thermo Fisher Scientific, Waltham, MA, USA). The qRT-PCR reaction conditions started at 95°C for 10 min, with the next 40 cycles at 95°C for 15 s, then 60°C for 45 s. The 2^−*ΔΔ*Ct^ method and normalization with *β*-actin were applied to determine the altered mRNA levels. Data are shown as the average of three repeats. The primers used are listed in Supplementary Table 1.

### 2.4. Western Blot

Total proteins prepared with RIPA lysis buffer (JRDUN, Shanghai, P.R. China) containing a protease inhibitor cocktail (P0013, Beyotime, China) were subjected to concentration determination using a BCA protein assay kit (P0012, Beyotime, China). Equal amounts of total protein (25 *μ*g) were fractionated with 10% SDS-PAGE (sodium dodecyl sulfate–polyacrylamide gel electrophoresis) and blotted to a nitrocellulose membrane (HATF00010, Millipore, Billerica, MA, USA) for overnight transfer. Following blockage by 5% nonfat dry milk for 1 hour at room temperature, the membranes were sequentially incubated overnight at 4°C with the primary antibodies SLC38A1 (Ab134268, Abcam, UK), HIF-1*α* (Ab1, Abcam, UK), p-mTOR (#5536, CST, USA), mTOR (#2972, CST, USA), and GAPDH (60004-1-1G, Proteintech, USA), and for 1 hour at 37°C with the secondary antimouse IgG antibody (1 : 1,000; Beyotime, Shanghai, P.R. China). An enhanced chemiluminescence system (Tanon-5200, Tanon, Shanghai, P.R. China) was used to develop the protein signal.

### 2.5. Cell Proliferation

We examined cell growth (2 × 10^5^/pore) at 0, 12, 24, and 48 hours with the Cell Counting Kit-8 (CCK8; CP002, SAB, USA). Briefly, after 1 hr incubation in the provided solution (1 : 10), treated cells underwent colorimetric analysis at a wavelength of 450 nm on a microplate reader (DNM-9602, Pulangxin, Beijing, P.R. China) to determine cell proliferation. Each experiment was carried out in triplicate.

### 2.6. Knockdown and Overexpression

A commercial company (Major Industrial Co., Ltd, Shanghai, China) synthesized short-hairpin RNA (shRNA) targeting human HIF-1*α*-1 (shHIF-1*α*-1; site 116–134; 5′-AGUUAUAGCUUCCCGACUATT-3′), shHIF-1*α*-2 (site 306–324; 5′- CCCACAAGUUCUUGUACUATT-3′), and shHIF-1*α*-3 (site 487–505; 5′- GAUUGUCA UGGACUUCUUATT-3′), as well as a negative control shRNA (shNC; 5′-UUCUCCGAACGUGUCACGUTT-3′). They then cloned these into lentiviral plasmids (pLKO.1). For overexpression, we used a standard procedure to create pLVX-puro lentiviral plasmids containing HIF-1*α* (NM_017902.3) or SLC38A1 (NM_001077484) cDNA, together with a mock plasmid negative control (oeNC). The cells were then introduced (2 × 10^5^/pore) with their corresponding plasmids using Lipofectamine 2000 and maintained for 48 hours before analysis.

### 2.7. Transwell Assay

Cells (1 × 10^5^/pore) inoculated in 24-well plates were kept in a 37°C and 5% CO_2_ incubator for 2 hours to ensure complete absorption of nutrients. After the inoculation of 1 × 10^5^ cells into the apical chamber, we supplemented up to 0.5 mL of 1% fetal bovine serum containing culture medium followed by 0.75 mL of 15% fetal bovine serum containing culture medium to the basolateral chamber. After incubating at 37°C with 5% CO_2_ for 20 hours, we discarded the culture medium in the apical chamber and subjected the cells to rinsing twice with phosphate-buffered saline, fixing in 4% paraformaldehyde for 10 min, washing twice more in phosphate-buffered saline for 2 min, and then staining with crystal violet for 10 min. After removing the Matrigel and cells using cotton swabs and rinsing three times with phosphate-buffered saline, we visualized and counted the cells in five randomly chosen fields using a light microscope (×200 magnification).

### 2.8. Dual Luciferase Reporter Gene Assay

Based on the predictions by TargetScan and Starbase, we produced DNA sequences containing either wild-type (WT) or mutant (Mut) miR-373-3p binding sites on the 3′-UTR of SLC38A1 and inserted these into the pGL3-Basic luciferase reporter vector to obtain the WT and Mut 3′-UTR constructs. Individual constructs, together with the miR-373-3p mimic, were co-introduced into HTR8 cells and the luciferase activities of these reporter constructs in receiving cells were determined 48 h after transfection, using a dual luciferase reporter gene kit (Beijing Yuanpinghao Biotechnology Co., Ltd.).

### 2.9. Statistical Analysis

We performed the statistical analyses in GraphPad Prism Version 7.0 (La Jolla CA, USA) and report all results as means and standard deviations for at least three samples or repeat experiments. Analyses were performed using *t*-tests and one-way analysis of variance for two groups and three or more groups, respectively. *p*-values of <0.05 denote statistical significance.

## 3. Results

### 3.1. HIF-1*α* Knockdown Boosted HTR8 Cell Migration and Growth in Response to Hypoxia

**Figure 1:**
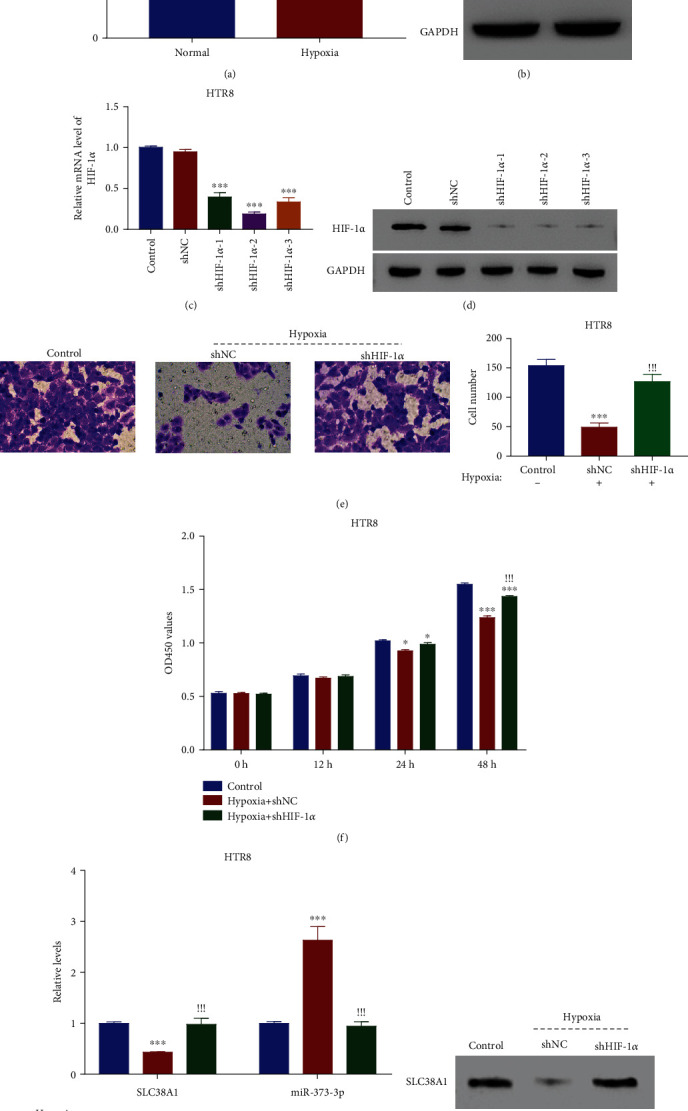
HIF-1*α* knockdown increased HTR8 cell migration and proliferation under hypoxic conditions. (a, b) qRT-PCR and western blot of the relative mRNA and protein levels of HIF-1*α* under normal and hypoxic conditions in human HTR8 cells. ^∗∗∗^*p* < 0.001 vs Normal. (c, d) HIF-1*α* shRNAs significantly suppressed HIF-1*α* expression in HTR8 cells. ^∗∗∗^*p* < 0.001 vs shNC. (e) Transwell assay of cell migration. ^∗∗∗^*p* < 0.001 vs shNC. ^!!!^*p* < 0.001 vs shNC. (f) CCK8 assay of the proliferation of HTR8 cells after transfecting shNC and shHIF-1*α* under hypoxia conditions. ^∗^*p* < 0.05 vs control, ^∗∗∗^*p* < 0.001 vs control; ^!!!^*p* < 0.001 vs hypoxia + shNC. (g) qRT-PCR of the relative mRNA levels of SLC38A1 and miR-373-3p in HTR8 cells after transfecting with shNC or shHIF-1*α* under hypoxic conditions. ^∗∗∗^*p* < 0.001 vs control; ^!!!^*p* < 0.001 vs hypoxia + shNC. (h) Western blot of SLC38A1 protein expression in HTR8 cells after transfecting with shNC or shHIF-1*α* under hypoxic conditions.

We initially evaluated the alterations of miR-373-3p and HIF-1*α* in HTR8 cells exposed to hypoxia. Figures 1(a) and 1(b) show that hypoxia not only markedly induced HIF-1*α* expression but also boosted miR-373-3p levels compared with normal conditions.

To validate the role of HIF-1*α* in HTR8 cell behavior, we reduced HIF-1*α* expression using a shRNA-mediated method. All three shRNAs targeting HIF-1*α* significantly reduced the expression of HIF-1*α*, as verified by the mRNA and protein levels, with shHIF-1*α*-2 presenting the strongest inhibitory action (Figures 1(c) and 1(d)). Analysis of migratory behavior by transwell assay under normal and hypoxic conditions then revealed that hypoxia suppressed HTR8 cell migration and that HIF-1*α* downregulation reversed this decrease robustly (Figure 1(e)). Cell growth analysis by CCK8 assay also showed that hypoxia repressed HTR8 cell proliferation at after 24 hours, whereas HIF-1*α* knockdown effectively attenuated this inhibitory effect (Figure 1(f)). Consistent with these alterations, HIF-1*α* knockdown almost completely abolished the hypoxia-induced upregulation of miR-373-3p and downregulation of SLC38A1 compared with the control (Figures 1(g) and 1(h)).

Collectively, HIF-1*α* responds effectively to a hypoxic challenge in HTR8 cells, suggesting the successful establishment of a cellular model with HIF-1*α* downregulation promoting HTR8 cell proliferation and migration and SLC38A1 gene expression.

### 3.2. HIF-1*α* Overexpression Abolished the Action of miR-373-3p Inhibitor in HTR8 Cells

**Figure 2:**
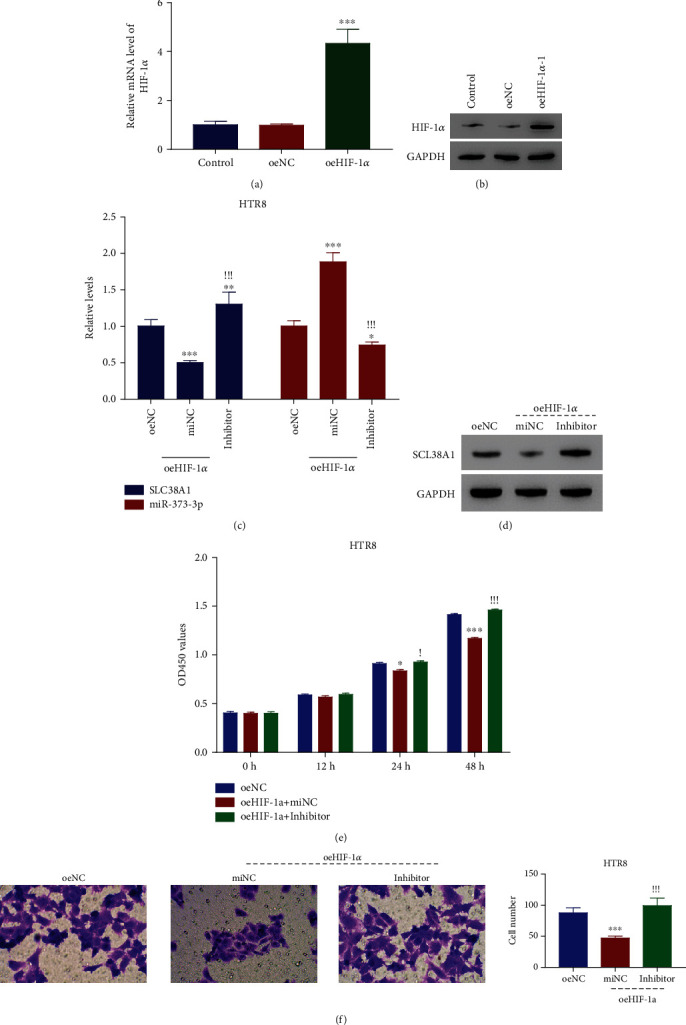
HIF-1*α* overexpression abolished miR-373-3p inhibitor function in HTR8 cells. (a, b) qRT-PCR and western blot of the relative HIF-1*α* mRNA and protein levels in HTR8 after transfecting with oeNC and oeHIF-1*α*. ^∗∗∗^*p* < 0.001 vs oeNC. (c) qRT-PCR of SCL38A1 protein levels in HTR8 cells treated with miR-373-3p miNC or inhibitor after transfecting with oeHIF-1*α*. ^∗^*p* < 0.05 vs oeNC, ^∗∗^*p* < 0.01 vs oeNC, ^∗∗∗^*p* < 0.001 vs oeNC. ^!!!^*p* < 0.001 vs miNC + oeHIF-1*α*. (d) Western blot of the SCL38A1 protein levels in HTR8 cells treated with miR-373-3p miNC or inhibitor after transfecting with oeHIF-1*α*. (e, f) The miR-373-3p inhibitor significantly promoted the proliferation and migration of HTR8 cells after transfecting with oeHIF-1*α*. ^∗^*p* < 0.05 vs oeNC, ^∗∗∗^*p* < 0.001 vs oeNC. ^!!!^*p* < 0.001 vs miNC + oeHIF-1*α*.

Providing robust evidence for the role of HIF-1*α* in mediating the action of miR-373-3p, lentiviral-mediated HIF-1*α* overexpression enhanced both mRNA and protein levels in HTR8 cells relative to oeNC transduced cells (Figures 2(a) and 2(b)). Subsequently, we treated HIF-1*α* overexpressed cells with a miR-373-3p inhibitor, and when compared to miNC treated cells (controls), this almost completely stopped the miR-373-3p upregulation and SCL38A1 downregulation (Figures 2(c) and 2(d)). The simultaneous presence of both a miR-373-3p inhibitor and HIF-1*α* overexpression markedly enhanced the proliferative and migratory capabilities of HRT8 cells relative to HIF-1*α* overexpression alone (Figures 2(e) and 2(f)). Thus, HIF-1*α* overexpression can abrogate the effects of a miR-373-3p inhibitor on cell behavior and gene expression.

### 3.3. miR-373-3p Restricted the Growth and Migration of HTR8 Cells

**Figure 3:**
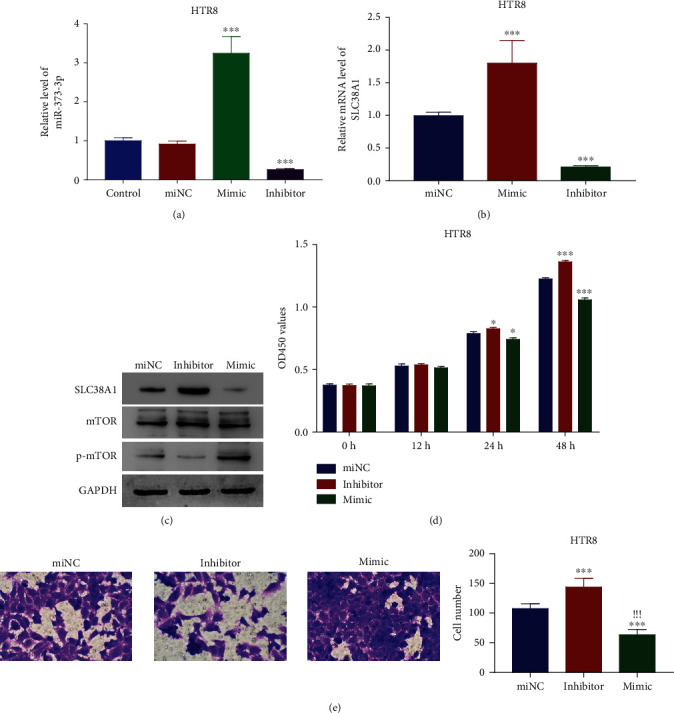
miR-373-3p suppressed HTR8 cell proliferation and migration. (a) miR-373-3p silencing and overexpression induced by the corresponding mimic and inhibitor. ^∗∗∗^*p* < 0.001 vs miNC. (b) qRT-PCR of the relative mRNA levels of SLC38A1 in HTR8 cells treated with miNC, miR-373-3p, and inhibitor. ^∗∗∗^*p* < 0.001 vs miNC. (c) Western blot of the protein levels of SLC38A1, mTOR, and p-mTOR in HTR8 cells treated with miNC, miR-373-3p, and inhibitor. (d) CCK8 assay of HTR8 cell proliferation after treatment with the miR-373-3p inhibitor or mimic. ^∗^*p* < 0.05 vs miNC, ^∗∗∗^*p* < 0.001 vs miNC. (e) Transwell assay of HTR8 cell migration after treatment with the miR-373-3p inhibitor or mimic. ^∗∗∗^*p* < 0.001 vs miNC; ^!!!^*p* < 0.001 vs inhibitor.

To address how miR-373-3p causes the phenotypic alterations of trophoblast cells, we used qRT-PCR and western blot to analyze the regulatory effects of a miR-373-3p mimic and inhibitor on miR-373-3p itself, its target gene SLC38A1, and related mTOR signaling. Comparing the mimic and inhibitor with their controls confirmed the respective upregulation and downregulation of miR-373-3p and the respective downregulation and upregulation of SLC38A1 (Figures 3(a)–3(c)). HRT8 cells exposed to the miR-373-3p inhibitor or mimic either inactivated or activated mTOR, respectively, relative to the control (Figure 3(c)). The downregulation of miR-373-3p by the inhibitor enhanced the proliferative and migratory capabilities of HRT8 cells, while its upregulation by the mimic exerted an inhibitory effect (Figures 3(d) and 3(e)). These findings indicate that miR-373-3p represses the proliferative and migratory behaviors of HRT8 cells.

### 3.4. Mir-373-3p Curbed SLC38A1 Expression by Interacting with Its 3′-UTR

**Figure 4:**
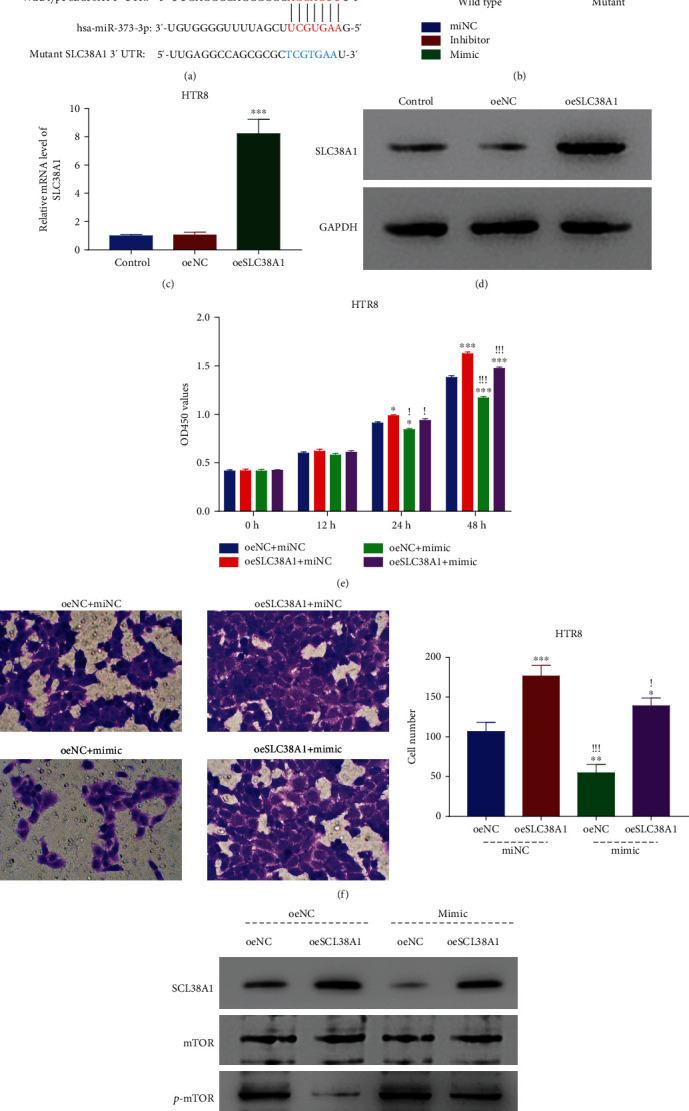
miR-373-3p inhibited SLC38A1 function by binding to its 3′-UTR. (a) WT and mutant binding sites of miR-373-3p in the 3′-UTR of SLC38A1. (b) Mutant 3′-UTR abolished the interaction between miR-373-3p and SLC38A1. ^∗∗∗^*p* < 0.001 vs miNC. (c, d) Lentiviral-mediated vector in HTR8 cells induced SLC38A1 overexpression. ^∗∗∗^*p* < 0.001 vs oeNC. (e) SLC38A1 overexpression significantly upregulated the proliferation of mimic treated cells. ^∗^*p* < 0.05 vs oeNC + miNC, ^∗∗∗^*p* < 0.05 vs oeNC + miNC, ^!^*p* < 0.05 vs oeSLC38A1 + miNC, ^!!!^*p* < 0.001 vs oeSLC38A1 + miNC. (f) The miR-373-3p mimic inhibited the migration of oeSLC38A1 transfected cells. ^∗^*p* < 0.05 vs oeNC + miNC, ^∗∗^*p* < 0.01 vs oeNC + miNC, ^∗∗∗^*p* < 0.001 vs oeNC + miNC. ^!^*p* < 0.05 vs oeSLC38A1 + miNC, ^!!!^*p* < 0.001 vs oeSLC38A1 + miNC. (g) Western blot of SCL38A1, mTOR, and p-mTOR protein levels in cells.

To clarify how miR-373-3p controls the expression, and thereby affects the function of SLC38A1, we generated firefly luciferase constructs containing either the WT or Mut miR-373-3p binding site on the 3′-UTR of the SLC38A1 gene, according to bioinformatic analysis (Figure 4(a)). The promoting and inhibiting actions of miR-373-3p on the WT construct's luciferase activity vanished for the Mut construct (Figure 4(b)). To further verify the link between those two molecules, we then enhanced the expression of SLA38A1 in HTR8 cells using a lentiviral-mediated method (oeSLC38A1). This greatly increased mRNA and protein levels of the SLA38A1 gene compared with the negative control and empty lentiviral transduced control (oeNC) (Figures 4(c) and 4(d)). The HTR8 cell growth was then analyzed with the oeSLA38A1 and the miR-373-3p mimic alone or simultaneously. This showed that individual SLA38A1 overexpression boosted the proliferative ability of HTR8 cells, while the single application of the mimic restricted cell growth relative to the control at both 24 and 48 hours after treatment. Simultaneous SLA38A1 overexpression with the miR-373-3p added rendered cell proliferation almost comparable to that of SLA38A1 overexpression alone, suggesting that the former abrogated the inhibitory action of the latter (Figure 4(e)). A similar phenomenon was observed for cell migration, with the promoting effect of SLA38A1 overexpression on cell invasion present even with the miR-373-3p mimic (Figure 4(f)). Accompanying these phenotypic alterations, mTOR activity was obviously repressed by either individual SLA38A1 overexpression or simultaneous SLA38A1 overexpression and miR-373-3p mimic application, when compared to the corresponding control. The forward and reverse evidence robustly confirms that miR-373-3p represses the function of SLA38A1 by targeting the expression of SLA38A1 through an interaction with its 3′-UTR.

### 3.5. The Placental Tissue of the Low Weight sIUGR Twins Had miR-373-3p Upregulation

To demonstrate the clinical relevance of miR-373-3p and SLC38A1 gene upregulation in sIUGR, we used qRT-PCR to analyze the miR-373-3p content of placental tissues from sIUGR twins (*n* =5) and normal monochorionic diamniotic twins (control, *n* =5) with low or high weight, respectively. The miR-373-3p content in the placental tissues of sIUGR twins with lower fetal weights was significantly higher than in those with heavier fetal weights, suggesting the upregulation of this miRNA under the sIUGR condition with smaller fetuses (Figure S1, A). The qRT-PCR and western blot results then revealed markedly decreased SLC38A1 mRNA and protein levels in the placental tissues from sIUGR twins with low fetus weights compared with the placentas with heavy fetal weights. The SLC38A1 expression levels did not differ obviously between larger and smaller fetus in the control group (Figure S1, A and B). Of note, we observed activated mTOR signaling, with higher phosphorylation levels and downstream gene upregulation (HIF-1*α*) evident in the placental tissues of light compared with heavy fetuses in the sIUGR group; however, the control group did not show such alterations (Figure S1, B). These results confirm the roles of miR-373-3p upregulation and SLC38A1 downregulation associated with an altered mTOR pathway in the progression of sIUGR.

## 4. Discussion

The exact pathogenesis of sIUGR in twin pregnancy remains unknown, though uneven placental sharing and vascular anastomoses appear relevant [4–6]. Given that monochorionic twins have identical genetic information, the role of epigenetic factors warrants serious consideration. Thus, we studied the role of miR-373-3p in modulating the growth and migration of trophoblast cells based on two key pieces of evidence: first, that smaller sIUGR fetuses have marked elevations of this miRNA in their placental tissues; second, that its upregulation or downregulation in trophoblast cells alters the growth and migratory behaviors of those cells.

Besides evidence of increased miR-373-3p levels in multiple cancers [34–37], research has demonstrated its upregulation in preterm labor placentas relative to normal placentas [13], a finding partially consistent with our observation in the placental tissues of smaller sIUGR fetuses. It is well-known that placentation relies heavily on the proliferative and migratory abilities of trophoblast cells [19]; therefore, any factors that alter these properties affect placental development. The observations by Lee and colleagues, together with our findings of increased expression in placental tissues and a regulatory action on trophoblast cells, robustly support the key role of miR-373-3p in placental development [13]. This further highlights the significance of deregulated miRNAs, such as miR-210, miR-376c, and miR-455 [38–40], as well as miR-373-3p validated in this study, in the development of placenta-related disorders. Notably, previous research demonstrated that miR-373-3p modulated trophoblast cell behavior by simultaneously targeting CD44 and RDX, thereby affecting adhesion- and migration-related downstream pathways [13]. By contrast, we now report that miR-373-3p exerted its effect on trophoblast cell growth and invasion by targeting the amino acid transporter SLC38A1 [8]. These findings support that miR-373-3p regulates multiple genes, even simultaneously [13, 41], and provide further evidence to show the contribution of this miRNA and other target genes in the progression of sIUGR.

Evidence from multiple sources now supports the existence of a regulatory connection between miR-373-3p and its target SLC38A1. In the current study, we report three main findings: first, smaller sIUGR fetuses had lower placental SLC38A1 expression, contrasting with the upregulation seen with miR-373-3p; second, introducing the miR-373-3p mimic or inhibitor to trophoblast cells led to SLC38A1 downregulation or upregulation, respectively; and third, we identified an interaction between miR-373-3p and the 3′-UTR of SLC38A1, experimentally verifying the regulation of the latter by the former. Other studies have also recognized the important contribution of SLC38A1 as an amino acid transporter in placental and fetal development [42–46]. Similarly, research has already described the relationship of SLC38A1 with miR-150-5p, miR-593-3p, and miR-4317 in specific pathological conditions [47–49]. However, no study has reported the regulatory action of miR-373-3p on SLC38A1 until now.

The placenta functions as an important interface for the exchange of nutrients, oxygen, and waste between the mother and fetus [19]. Active trophoblast cell growth, differentiation, and migration are important to placental development and may be restricted by hypoxia [50, 51]. HIF-1*α* and mTOR are key mediators of many metabolic processes that occur in response to cell hypoxia [52]. The present study HIF-1*α* and mTOR are key mediators of many metabolic processes that occur in response to cell hypoxia [52], and consistent with the changes in miR-373-3p and SLC38A1, we uncovered higher mTOR activity and HIF-1*α* levels in the placental tissues of smaller sIUGR fetuses. Likewise, HIF-1*α* downregulation enhanced the proliferation and migration of trophoblast cells and the expression of SLC38A1, while the altered miR-373-3p and SLC38A1 levels influenced both their own functions and the activities of mTOR and HIF-1*α* in trophoblast cells, suggesting an interplay between the miR-373-3p/SLC38A1 axis and mTOR/HIF1*α* signaling.

In summary, the placental tissues of smaller sIUGR fetuses and trophoblast cells exposed to hypoxia showed miR-373-3p upregulation associated with decreased trophoblast cell growth and invasion. We revealed SLC38A1 as the likely target effector of miR-373-3p and showed the role of the miR-373-3p/SLC38A1 axis in not only regulating trophoblast cell behavior but also in mTOR/HIF-1*α* signaling. Therefore, our findings highlight the vital modulatory role of miR-373-3p and its downstream gene *SLC38A1* in placental and fetal development, providing further evidence that epigenetic factors affect the pathogenesis of sIUGR. They also suggest the potential of the miR-373-3p/SLC38A1 axis as a new target for diagnosing and treating sIUGR. However, because we obtained the in vitro data from observations in a single cell line, we must now rectify this limitation by confirming these results in other cell lines.

## Data Availability

Data would be available on request to corresponding author.

## Supplementary Materials

Supplementary 1 Supplementary Table 1: Primer sequence information.

Supplementary 2 Supplementary Figure S1: miR-373-3p was upregulated in the placenta tissues of low weight from sIUGR twin. (a, b) qRT-PCR was used to examine the relative levels of miR-373-3p and SLC38A1 in the placenta tissues of high or low weight from normal and sIUGR twin. ∗∗∗*p* < 0.001 vs high weight. (c) Western blot was used to examine the protein levels of SLC38A1, mTOR, p-mTOR, and HIF-1*α* in the tissues as indicated above.
