# Supplementary material for: miR-373-3p Regulates the Proliferative and Migratory Properties of Human HTR8 Cells via SLC38A1 Modulation
Source: Dis Markers. 2022 Jun 28;2022:6582357. doi: 10.1155/2022/6582357 (PMC9274228; doi:10.1155/2022/6582357)
Supplement: Supplementary 1 — Supplementary Table 1: Primer sequence information. [file 6582357.f1.docx]

Supplementary Table 1

| Name | Forward | Reverse |
| --- | --- | --- |
| has-miR-373-3p | 5'- AACAAGGAAGTGCTTCGATTTTGG-3' | 5'-CAGTGCAGGGTCCGAGGT-3' |
| U6 | 5'-CTCGCTTCGGCAGCACA-3' | 5'-AACGCTTCACGAATTTGCGT-3' |
| SLC38A1 | 5'-TGACAGTGCCCGAGGATGATA-3' | 5'-AGACATGCCTAAGGAGGTTGTA-3' |
| GAPDH | 5'-AATCCCATCACCATCTTC-3' | 5'-AGGCTGTTGTCATACTTC-3' |
