# Supplementary material for: miR-373-3p Regulates the Proliferative and Migratory Properties of Human HTR8 Cells via SLC38A1 Modulation
Source: Dis Markers. 2022 Jun 28;2022:6582357. doi: 10.1155/2022/6582357 (PMC9274228; doi:10.1155/2022/6582357)
Supplement: Supplementary 2 — Supplementary Figure S1: miR-373-3p was upregulated in the placenta tissues of low weight from sIUGR twin. (a, b) qRT-PCR was used to examine the relative levels of miR-373-3p and SLC38A1 in the placenta tissues of high or low weight from normal and sIUGR twin. ∗∗∗p < 0.001 vs high weight. (c) Western blot was used to examine the protein levels of SLC38A1, mTOR, p-mTOR, and HIF-1α in the tissues as indicated above. [file 6582357.f2.docx]

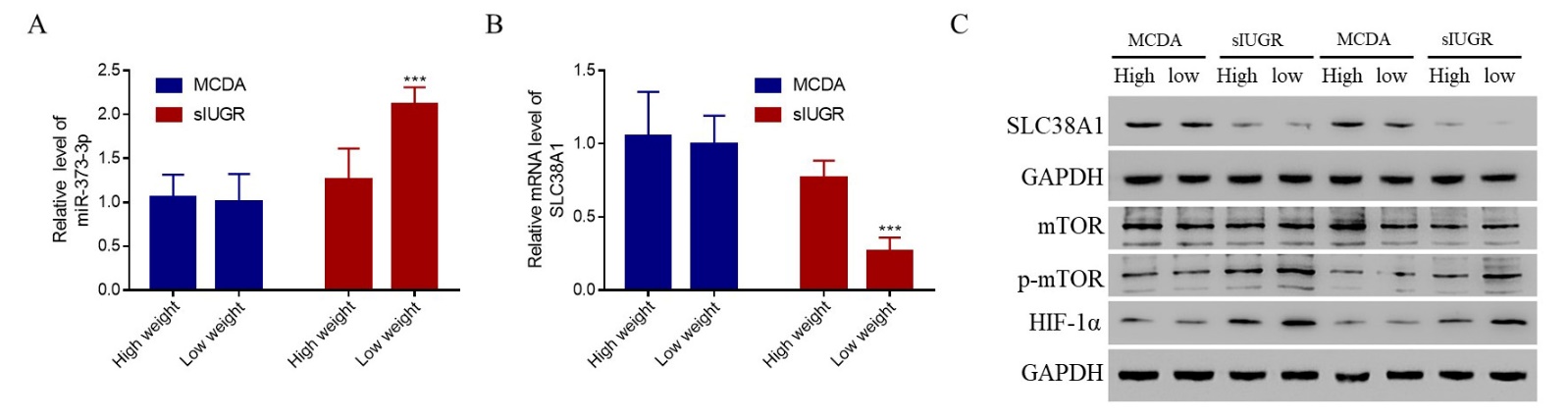


Figure S1: miR-373-3p was upregulated in the placenta tissues of low weight from sIUGR twin. A and B. qRT-PCR was used to examine the relative levels of miR-373-3p and SLC38A1 in the placenta tissues of high or low weight from normal and sIUGR twin. *** p<0.001 vs high weight. C. Western blot was used to examine the protein levels of SLC38A1, mTOR, p-mTOR and HIF-1α in the tissues as indicated above.
